# Association of lymphocyte-to-C-reactive protein ratio with cerebral small vessel disease: a cross-sectional study based on dose-response analysis

**DOI:** 10.3389/fneur.2024.1480115

**Published:** 2024-12-24

**Authors:** Jie Lin, Junyi Liu, Qian Luo, Jieying Zhuang, Ruiyan Xiao, Huijuan Wang, Xudong Yang, Xiaolan Wei, Jiangping Cai

**Affiliations:** Department of Neurology, The First Hospital of Quanzhou Affiliated to Fujian Medical University, Quanzhou, Fujian, China

**Keywords:** inflammation, cerebral small vessel disease, biomarkers, lymphocyte-to-C-reactive protein ratio, cross-sectional study

## Abstract

**Objective:**

We investigated the relationship between lymphocyte-to-C-reactive protein ratio (LCR) and common imaging markers of cerebral small vessel disease (CSVD).

**Methods:**

Data from 835 CSVD patients were analyzed using univariate and multivariate logistic regression to determine CSVD-associated factors. Multivariate models assessed the association between LCR and CSVD, including common imaging markers. Subgroup analysis by age, sex, smoking history, hypertension, lipid levels, and other factors was conducted. The receiver operating characteristic curve analysis and 10-fold cross-validation were performed to evaluate the predictive performance of LCR.

**Results:**

Lymphocyte-to-C-reactive protein ratio was independently associated with a decreased risk of CSVD (*p* < 0.001), indicating a protective role of LCR against CSVD. Among the imaging markers of CSVD, LCR in the highest quartile was negatively associated with moderate-to-severe white matter hyperintensities (WMH) (*p* = 0.002) and moderate-to-severe enlarged perivascular spaces (EPVS) (*p* < 0.001), but not with lacune (*p* > 0.05). The restrictive cubic spline analysis revealed a linear dose-response relationship between log-transformed LCR and the incidence of CSVD (*P*_non-linear_ = 0.090) as well as moderate-to-severe WMH (*P*_non-linear_ = 0.304), with a non-linear association with moderate and severe EPVS (*P*_non-linear_ = 0.001). In the subgroup analyses, LCR remained a significant association with CSVD in most subgroups (*p* < 0.05). Notably, a significant correlation was observed between LCR and CSVD (*p* < 0.001) in the subgroups of non-smokers, those with neutrophil count ≤6.3 × 10^9^/L, and with high-density lipoprotein cholesterol ≥1 mmol/L. No interaction effect was identified between the variables and the LCR (*p* > 0.1). The predictive capability of LCR for CSVD was confirmed through receiver operating characteristic curve analysis.

**Conclusion:**

Lymphocyte-to-C-reactive protein ratio is an independent protective factor for CSVD and is associated with lower WMH or EPVS burden but not lacune. Inflammation is involved in CSVD pathophysiology through multiple pathways, providing potential targets for CSVD intervention.

## 1 Introduction

Cerebral small vessel disease (CSVD) comprises a range of pathological syndromes affecting the microvascular system of the brain. Characteristic imaging manifestations of CSVD comprise recent small subcortical infarctions, enlarged perivascular spaces (EPVS), white matter hyperintensities (WMH), and cerebral microbleeds (1, 2). The incidence of CSVD is significantly correlated with age. Inflammaging, which has attracted increasing attention in recent years, refers to a chronic, aseptic, low-grade proinflammatory state triggered by the increase of systemic inflammation and peripheral immune senescence. It serves as a common underlying factor for various conditions such as cardiovascular disease, stroke, neurodegenerative diseases, and cancer (3–8). An increasing body of evidence (9, 10) suggests that inflammaging plays a role in the pathophysiological mechanisms of CSVD, altering the blood–brain barrier permeability through the initiation of a molecular cascade and an aberrant inflammatory reaction. This process causes the release of antigens from the central nervous system into the peripheral circulation, and lymphocytes infiltrating the brain tissue, resulting in associated brain damage. Moreover, brain damage can further compromise the immune system, creating a detrimental cycle (11–13). Therefore, investigating the relationship between inflammation and CSVD may provide insights into the pathogenesis of this condition.

Different inflammatory markers are implicated in various inflammatory pathways of CSVD. A study examining multiple circulating markers of inflammation revealed a significant association between tumor necrosis factor-*α*, peroxidase, and cerebral microbleeds (14). Elevated baseline homocysteine (Hcy) levels have been extensively studied concerning CSVD, showing a correlation with its development and imaging burden (15, 16). A longitudinal cohort demonstrated that systemic inflammation levels measured by fibrinogen and C-reactive protein (CRP) were associated with an increased risk of vascular dementia (17). Furthermore, various proinflammatory factors including soluble vascular calcium adhesion molecule-1, thrombin-antithrombin, and IL-6 were independently associated with lacunar stroke (18, 19). In addition, new indicators of systemic inflammation, including the neutrophil-lymphocyte ratio (NLR) and systemic immune-inflammatory index, have been developed based on complete blood count ratios and have shown to be valuable in evaluating CSVD and its severity (20–22). However, the specific associations between various inflammatory biomarkers and the development of CSVD remain poorly understood.

The lymphocyte-to-C-reactive protein ratio (LCR) is a cost-effective and convenient composite measure of inflammation, first proposed by Okugawa et al. (23). The calculation involves dividing the lymphocyte count by the CRP concentration. By utilizing this ratio instead of considering only the lymphocyte count or CRP concentration separately, it can help adjust for confounding factors affecting biochemical parameters, thereby enhancing the sensitivity and reliability of predictive outcomes (24). Recent studies have indicated that LCR serves as a valuable prognostic indicator for patients facing cardiovascular adverse events, malignant tumors, undergoing hemodialysis, or dealing with coronavirus pneumonia (25–29). Despite these findings, studies on the relationship between LCR and cerebrovascular disease, especially CSVD, remain limited.

The term “dose-response relationship,” traditionally linked to toxicology and pharmacology, has been expanded in recent studies (30–34) to include the magnitude of various indicators, such as biomarker concentration and treatment intensity. In our study, we applied this concept to investigate the relationship between LCR (interpreted as an “inflammatory dose”) and the severity of CSVD (interpreted as a “response”), along with its typical imaging markers, to assess any potential dose-response relationship. Our findings may contribute to a deeper understanding of the pathogenesis of CSVD and facilitate the exploration of potential targets for intervention.

## 2 Materials and methods

### 2.1 Participants and ethics

This retrospective study enrolled outpatients from the Department of Neurology, Quanzhou First Hospital Affiliated to the Fujian Medical University from February 2018 to July 2021. All participants underwent 3.0T cranial magnetic resonance imaging (MRI), blood routine, and biochemical tests. Inclusion criteria: (1) patients aged 36–85 years; (2) patients underwent cranial MRI, which included T1-weighted MRI, T2-weighted MRI, fluid-attenuated inversion-recovery imaging, and diffusion-weighted MRI; (3) serum CRP, Hcy, and other biochemical markers were detected at least 8 h after fasting. Exclusion criteria: (1) Patients with a history of previous large-vessel occlusion or other large-area cerebral infarction that could interfere with the diagnosis of CSVD; (2) patients with severe stenosis or occlusion in major cerebral vessels on CT angiography or digital subtraction angiography; (3) patients with contraindications to MRI examination; (4) patients with severe systemic inflammatory conditions within the past 2 weeks, including hematological disorders, history of surgery or severe trauma and active infection; (5) patients with an active COVID-19 infection or a history of COVID-19 infection; (6) patients on immunosuppressants or glucocorticoids within the past 2 weeks; (7) patients with a history of acute subarachnoid hemorrhage, cerebrovascular malformation, aneurysm subarachnoid hemorrhage, or presence of an untreated aneurysm (diameter > 3 mm); (8) patients with an expected survival of less than 5 years due to severe organic diseases, such as malignancy or severe renal failure; (9) patients with neurodegeneration diseases such as Parkinson’s disease or Alzheimer’s disease; (10) patients with significant non-vascular white matter lesions, such as leukospinal sclerosis and metabolic encephalopathy; and, (11) patients with incomplete clinical data. This study was performed in line with the principles of the Declaration of Helsinki. Approval was granted by the Ethics Committee of Quanzhou First Hospital Affiliated to the Fujian Medical University. All participants signed written informed consent.

### 2.2 Clinical data collection

Baseline clinical data on demographics and cardiovascular risk factors were collected through interviews and questionnaires at the time of enrollment. This included information on sex, age, height, weight, smoking, drinking, hypertension, diabetes, coronary heart disease, stroke, etc. Hypertension was defined as a blood pressure reading of 140/90 mmHg or higher without the use of antihypertensive medication, or with the use of such medication. Diabetes was diagnosed based on a fasting glucose level of ≥7 mmol/L, a 2-h glucose level of ≥11.1 mmol/L on an oral glucose tolerance test, or the patient being on anti-diabetic medication. The presence of coronary heart disease and stroke was validated by reviewing past medical records.

### 2.3 Laboratory assessment

All participants underwent venous blood sampling after a minimum 8-h fasting period. Various parameters including white blood cell count, neutrophil count (NC), lymphocyte count, full-range CRP, platelet count, albumin (Alb), low-density lipoprotein cholesterol (LDL-C), high-density lipoprotein cholesterol (HDL-C), Hcy, fasting plasma glucose (FPG), uric acid (UA), and estimated glomerular filtration rate (eGFR) were measured. The full-range CRP kit (Lifotronic, Shenzhen, China), which is based on the immunonephelometric method, was used to detect the level of full-range CRP. The detection was conducted on an automatic Lifotronic PA990 analyzer (Lifotronic). The lymphocyte-to-CRP ratio (LCR) was calculated.

### 2.4 MRI evaluation

Cranial MRI images of patients were evaluated by two trained neurologists in a blinded manner, following the STandards for ReportIng Vascular changes on nEuroimaging (35). The Fazakes rating scale (36) was used to evaluate the severity of periventricular white matter hyperintensity (PWMH) and deep white matter hyperintensity (DWMH), with mild scores ranging from 0 to 1 and moderate to severe scores from 2 to 3. The total WMH burden was determined by summing the Fazekas scores of PWMH and DWMH, where mild was defined as scores from 0 to 2 and moderate to severe as scores from 3 to 6. A lacune was characterized as a round or ovoid hyperintense subcortical lesion measuring between 3 mm and 15 mm. EPVS were identified as linear or rounded lesions, typically smaller than 3 mm in diameter, displaying similar signal intensity to cerebrospinal fluid on both T1WI and T2WI images. The presence of enlarged perivascular spaces in the basal ganglia (BG-EPVS) and centrum semiovale (CSO-EPVS) was graded visually (37): None (0), 1–10 EPVS (1), 11–20 EPVS (2), 21–40 EPVS (3), and >40 EPVS (4). To express the severity of EPVS more efficiently, EPVS burden was categorized as mild (grades 0–1) and moderate–severe (grades 2–4).

### 2.5 Statistical analysis

Statistical analysis was conducted using R software version 4.3.1 and Python version 3.13.0. Continuous variables are described as mean ± SD or median (interquartile range), and categorical variables are presented as frequencies (percentages). Group differences were assessed through various statistical tests including the Kruskal–Wallis, Mann–Whitney *U*, Chi-square, and Fisher’s exact tests. Violin plots, which were generated using the “ggplot2” package and the “gghalves” package in R software, were used to evaluate the distribution and differences of LCR. The influencing factors of CSVD were determined by univariate and multivariate logistic regression analyses. A multivariate model incorporating categorical and continuous variables was used to examine the relationship between LCR, CSVD, and common imaging markers. Within the categorical model, LCR values were categorized into quartiles, with the median value for each quartile utilized as a continuous variable to examine linear trends. In the continuous model, the Log2-transformation of LCR values was implemented because of the non-normal distribution of LCR. Age and sex were adjusted in Model 1. Model 2 included adjustments for age, sex, body mass index (BMI), smoking, alcohol consumption, hypertension, diabetes, coronary heart disease, and stroke. Additionally, Model 3 further adjusted for age, sex, BMI, smoking, drinking, hypertension, diabetes, coronary heart disease, stroke, NC, Alb, FPG, LDL-C, HDL-C, Hcy, eGRF, and UA. The dose-response relationship was further analyzed using a restrictive cubic spline (RCS) analysis (“rcssci” package in R software) with nodes set to the 10th, 50th, and 90th percentiles, with reference points set at the 10th percentile. Participants were stratified into subgroups based on age, sex, smoking history, hypertension history, and various biomarker levels (LDL-C, HDL-C, Alb, FPG, Hcy, GFR, and UA). Results from the subgroup and interaction analyses were visually represented using forest plots, which were generated using the “jstable” package, the “grid” package, and the “forestploter” package in R software. The receiver operating characteristic (ROC) curve was plotted using the “pROC” package in R software to evaluate the predictive value of LCR and Model 3 for CSVD. The area under the ROC curve (AUC) was calculated and the optimal cut-off value was obtained. The ROC curves after 10-fold cross-validation were further obtained to evaluate the model’s predictive performance through Python and its commands. The dataset was divided into 10 equal-sized folds. Nine folds were used as training data to construct the model, and the remaining 1 fold was used as test data to assess the model’s performance. The whole process was repeated 10 times and the average values of the 10 results were obtained. A two-tailed *p* value <0.05 was considered statistically significant.

## 3 Results

### 3.1 Characteristics of the study population

**Figure 1:**
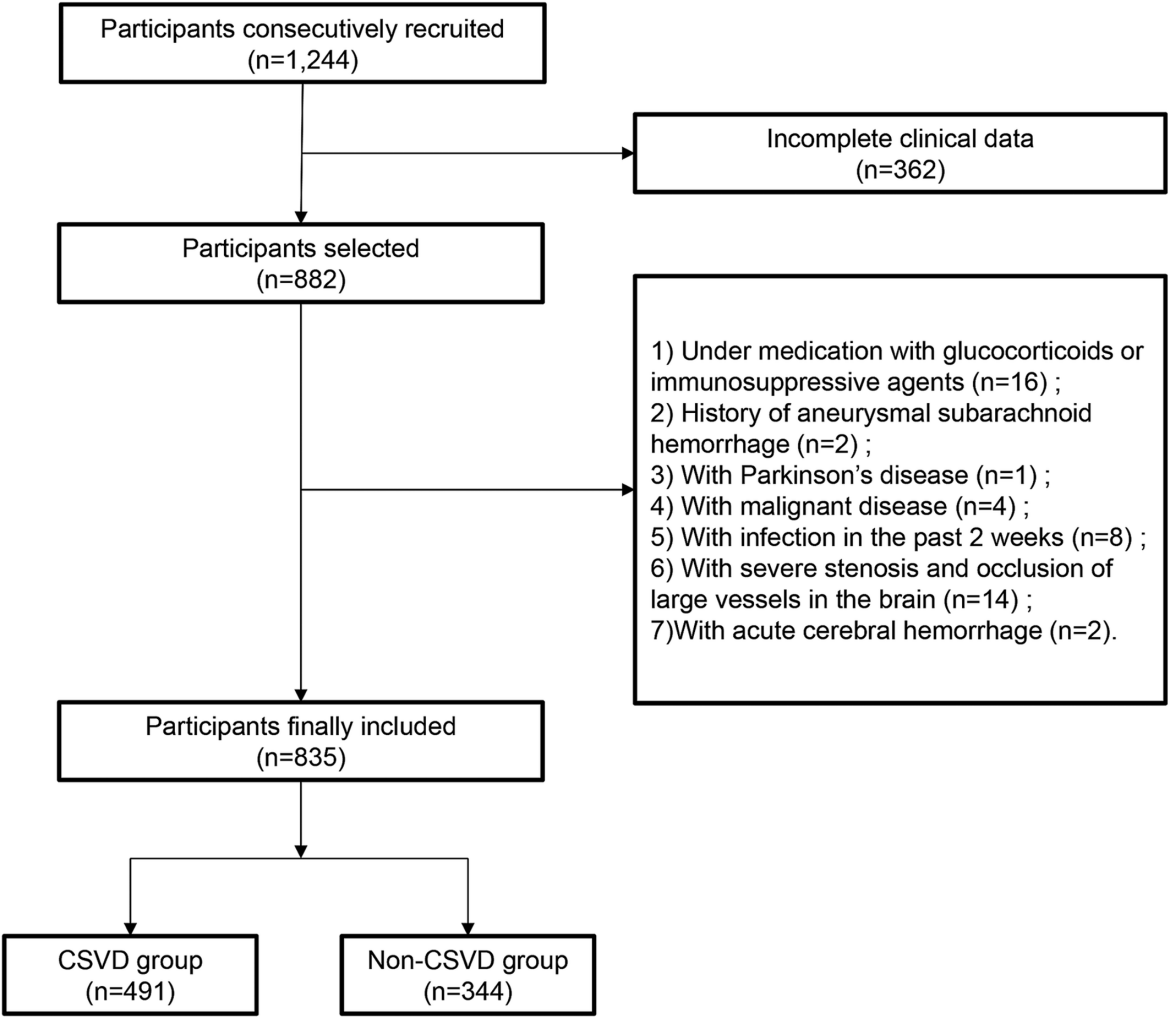
Flowchart of the study population enrollment.

In total, 835 patients were included in the study, with 491 (58.80%) diagnosed with CSVD (Figure 1). The baseline characteristics of the participants are presented in Table 1. The median age was 63.0 years (range 52.0–70.0) and there were 487 (58.32%) male participants. Patients were divided into Q1, Q2, Q3, and Q4 groups according to the quartiles of LCR values. There were significant differences in age, systolic blood pressure (SBP), hypertension, diabetes, coronary heart disease, stroke, NC, lymphocyte count, CRP, Alb, FPG, Hcy, eGFR, and UA among the groups (*p* < 0.05). The incidence of CSVD and the imaging burden were lower in the high quartile group compared to the low quartile group (*p* < 0.001).

**Table 1:** Characteristics of the study population.

Variable	Total	Quartiles of LCR				*P-*value
		Q1 (0.22, ≤0.42)	Q2 (0.68, 0.42–1.09)	Q3 (2.31, 1.09–3.25)	Q4 (4.19, >3.25)	
	(*N* = 835)	(*n* = 211)	(*n* = 207)	(*n* = 209)	(*n* = 208)	
Age (years)	63.00 (52.00–70.00)	67.00 (57.00–73.00)	63.00 (52.00–70.00)	62.00 (52.00–69.00)	57.00 (49.00–67.00)	<0.001
Sex, *n* (%)						0.230
Female	348 (41.68)	86 (40.76)	79 (38.16)	84 (40.19)	99 (47.60)	
Male	487 (58.32)	125 (59.24)	128 (61.84)	125 (59.81)	109 (52.40)	
BMI (kg/m^2^)	23.46 (22.04–24.77)	23.42 (22.04–24.78)	23.61 (22.43–24.94)	23.59 (22.03–24.75)	23.24 (21.98–24.50)	0.363
SBP (mmHg)	137.00 (122.50–156.00)	140.00 (126.00–158.00)	138.00 (125.00–152.00)	138.00 (120.00–159.00)	132.00 (118.00–149.00)	0.006
DBP (mmHg)	85.00 (76.00–95.00)	84.00 (76.00–94.00)	85.00 (78.00–97.00)	85.00 (75.00–95.00)	83.00 (75.00–94.00)	0.299
Smoking status, *n* (%)						0.313
Former and current	577 (69.1)	135 (63.98)	146 (70.53)	147 (70.33)	149 (71.63)	
Never	258 (30.9)	76 (36.02)	61 (29.47)	62 (29.67)	59 (28.37)	
Drinking status, *n* (%)						0.770
Former and current	726 (86.95)	182 (86.26)	180 (86.96)	179 (85.65)	185 (88.94)	
Never	109 (13.05)	29 (13.74)	27 (13.04)	30 (14.35)	23 (11.06)	
Hypertension, *n* (%)	410 (49.1)	118 (55.92)	112 (54.11)	92 (44.02)	88 (42.31)	0.007
Diabetes, *n* (%)	124 (14.85)	44 (20.85)	33 (15.94)	26 (12.44)	21 (10.10)	0.012
History of CHD, *n* (%)	59 (7.07)	23 (10.90)	16 (7.73)	11 (5.26)	9 (4.33)	0.041
History of stroke, *n* (%)	102 (12.22)	40 (18.96)	29 (14.01)	18 (8.61)	15 (7.21)	<0.001
White blood cell (10^9^/L)	7.00 (5.78–8.60)	7.28 (5.72–9.17)	7.15 (5.90–8.39)	6.91 (5.61–8.36)	6.71 (5.87–8.37)	0.280
Neutrophils (10^9^/L)	4.31 (3.26–5.92)	4.82 (3.52–6.74)	4.36 (3.38–5.55)	4.40 (3.24–5.96)	3.95 (3.11–5.05)	<0.001
Platelets (10^9^/L)	232.00 (197.00–275.00)	233.00 (185.00–281.50)	232.00 (193.50–274.50)	226.00 (197.00–266.00)	235.50 (205.75–278.75)	0.116
Lymphocytes (10^9^/L)	1.74 (1.33–2.20)	1.49 (1.04–1.83)	1.85 (1.49–2.29)	1.47 (1.17–1.84)	2.06 (1.83–2.54)	<0.001
CRP (mg/L)	1.89 (0.50–4.13)	6.39 (5.00–12.78)	2.56 (2.25–3.33)	0.52 (0.50–1.05)	0.50 (0.48–0.52)	<0.001
Albumin (g/L)	39.20 (37.10–41.80)	38.40 (36.20–40.60)	39.50 (37.40–42.85)	39.30 (37.30–42.20)	39.55 (37.50–41.52)	<0.001
FPG (mmol/L)	5.21 (4.75–5.92)	5.45 (4.87–6.31)	5.15 (4.71–5.87)	5.09 (4.68–5.81)	5.17 (4.71–5.68)	0.002
LDL-C (mmol/L)	3.14 (2.49–3.77)	3.05 (2.42–3.70)	3.18 (2.55–3.79)	3.12 (2.42–3.74)	3.24 (2.55–3.80)	0.314
HDL-C (mmol/L)	1.14 (0.98–1.36)	1.09 (0.95–1.31)	1.15 (0.98–1.37)	1.15 (0.96–1.35)	1.15 (1.02–1.42)	0.055
Homocysteine, μmol/L	10.50 (7.90–14.20)	12.40 (9.10–16.10)	10.60 (7.70–14.25)	10.30 (7.70–13.60)	9.45 (7.45–12.30)	<0.001
eGFR (mL/min × 1.73 m^2^)	96.08 (82.72–106.73)	91.65 (76.17–102.77)	95.01 (79.81–107.06)	97.20 (84.03–106.89)	101.84 (88.29–109.35)	<0.001
Uric acid, μmol/L	344.00 (282.50–423.50)	346.00 (282.00–422.50)	356.00 (292.50–442.50)	343.00 (289.00–409.00)	329.50 (270.75–417.25)	0.032
CSVD, *n* (%)	491 (58.8)	164 (77.73)	132 (63.77)	116 (55.50)	79 (37.98)	<0.001
WMH burden, *n* (%)						<0.001
Mild	416 (49.82)	66 (31.28)	97 (46.86)	114 (54.55)	139 (66.83)	
Moderate to severe	419 (50.18)	145 (68.72)	110 (53.14)	95 (45.45)	69 (33.17)	
PWMH burden, *n* (%)						<0.001
Mild	460 (55.09)	80 (37.91)	106 (51.21)	125 (59.81)	149 (71.63)	
Moderate to severe	375 (44.91)	131 (62.09)	101 (48.79)	84 (40.19)	59 (28.37)	
DWMH burden, *n* (%)						<0.001
Mild	516 (61.8)	97 (45.97)	124 (59.90)	137 (65.55)	158 (75.96)	
Moderate to severe	319 (38.2)	114 (54.03)	83 (40.10)	72 (34.45)	50 (24.04)	
EPVS burden, *n* (%)						<0.001
Mild	601 (71.98)	122 (57.82)	144 (69.57)	159 (76.08)	176 (84.62)	
Moderate to severe	234 (28.02)	89 (42.18)	63 (30.43)	50 (23.92)	32 (15.38)	
BG-EPVS burden, *n* (%)						<0.001
Mild	595 (71.26)	136 (64.45)	131 (63.29)	152 (72.73)	176 (84.62)	
Moderate to severe	240 (28.74)	75 (35.55)	76 (36.71)	57 (27.27)	32 (15.38)	
CSO-EPVS burden, *n* (%)						<0.001
Mild	497 (59.52)	91 (43.13)	110 (53.14)	130 (62.20)	166 (79.81)	
Moderate to severe	338 (40.48)	120 (56.87)	97 (46.86)	79 (37.80)	42 (20.19)	
Lacune, *n* (%)						<0.001
Yes	622 (74.49)	137 (64.93)	148 (71.50)	163 (77.99)	174 (83.65)	
No	213 (25.51)	74 (35.07)	59 (28.50)	46 (22.01)	34 (16.35)	

LCR, lymphocyte-to-C-reactive protein ratio; BMI, body mass index; SBP, systolic blood pressure; DBP, diastolic blood pressure; CHD history, coronary heart disease history; CRP, C-reactive protein; FPG, fasting plasma glucose; LDL-C, low-density lipoprotein cholesterol; HDL-C, high-density lipoprotein cholesterol; eGFR, estimated glomerular filtration rate; CSVD, cerebral small vessel disease; WHM, white matter hyperintensity; PWMH, periventricular white matter hyperintensity; DWMH, deep white matter hyperintensity; EPVS, enlarged perivascular spaces; BG-EPVS, basal ganglia enlarged perivascular spaces; CSO-EPVS, centrum semiovale enlarged perivascular spaces.

**Figure 2:**
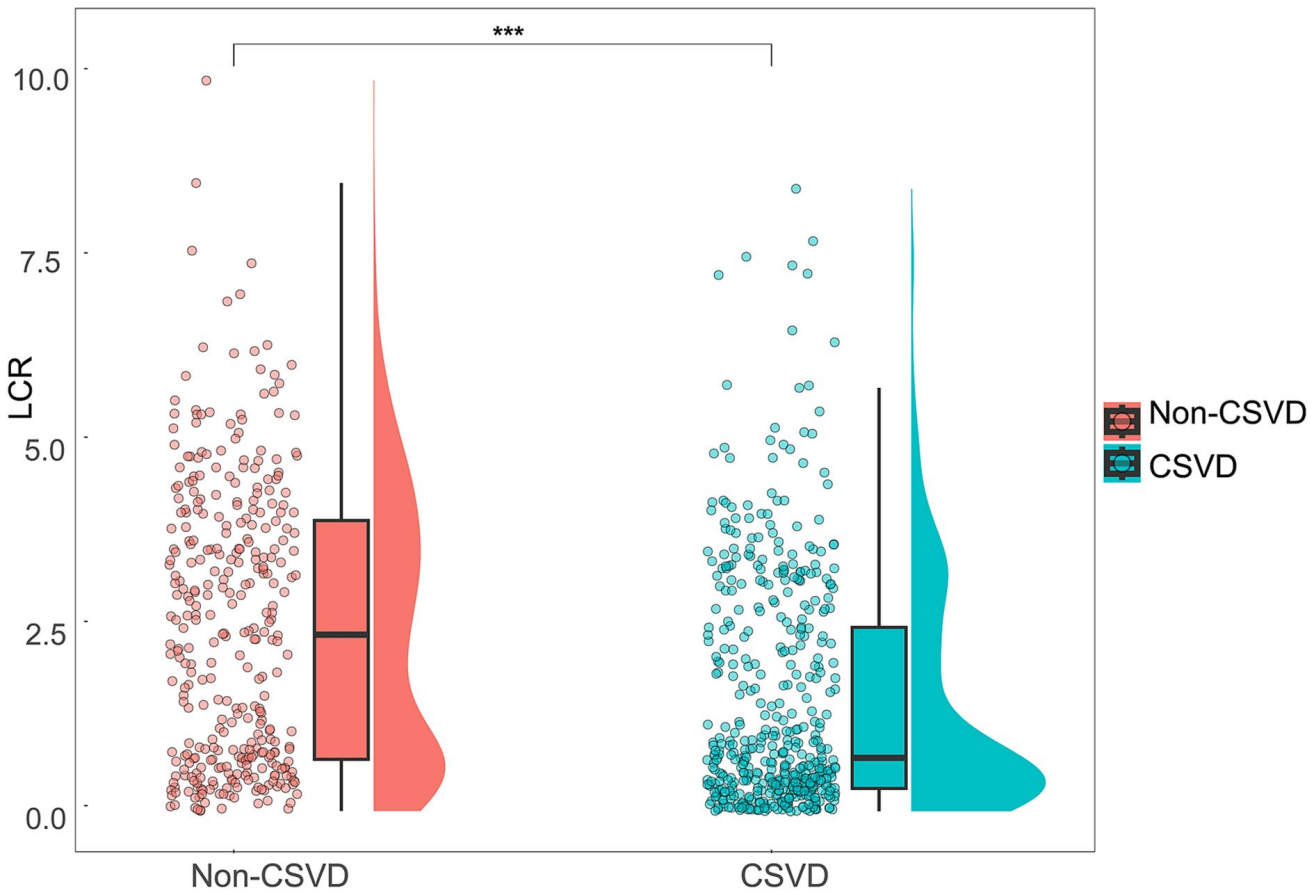
Distribution and comparison of LCR between the CSVD group and the non-CSVD group. Violin plots with scatter points and boxes were used to visualize the distribution and differences of LCR between the CSVD group and the non-CSVD group. ***p < 0.001.

Participants were further categorized into the CSVD group (*n* = 491) and the non-CSVD group (*n* = 344) (Table 2). There were significant differences in age, sex, SBP, history of hypertension, diabetes, coronary heart disease, stroke, NC, lymphocyte counts, CRP, Alb, FPG, LDL-C, HDL-C, Hcy, eGFR, and UA between CSVD and non-CSVD groups (*p* < 0.05). Notably, the CSVD group had significantly lower LCR than the non-CSVD group (*p* < 0.001) (Figure 2).

**Table 2:** Comparison between CSVD group and non-CSVD group.

Variable	Total	Non-CSVD	CSVD	*P*-value
	(*N* = 835)	(*n* = 344)	(*n* = 491)	
Age (years)	63.00 (52.00–70.00)	53.00 (47.00–63.00)	67.00 (59.00–73.00)	<0.001
Sex, *n* (%)				<0.001
Female	348 (41.68)	186 (54.07)	162 (32.99)	
Male	487 (58.32)	158 (45.93)	329 (67.01)	
BMI (kg/m^2^)	23.46 (22.04–24.77)	23.27 (22.03–24.77)	23.61 (22.13–24.78)	0.158
SBP (mmHg)	137.00 (122.50–156.00)	125.00 (114.75–138.00)	147.00 (132.00–165.50)	<0.001
DBP (mmHg)	85.00 (76.00–95.00)	80.00 (74.00–90.00)	88.00 (79.00–98.00)	<0.001
Smoking status, *n* (%)				<0.001
Never	258 (30.9)	49 (14.24)	209 (42.57)	
Former and current	577 (69.1)	295 (85.76)	282 (57.43)	
Drinking status, *n* (%)				<0.001
Never	109 (13.05)	19 (5.52)	90 (18.33)	
Former and current	726 (86.95)	325 (94.48)	401 (81.67)	
Hypertension, *n* (%)	410 (49.1)	82 (23.84)	328 (66.80)	<0.001
Diabetes, *n* (%)	124 (14.85)	11 (3.20)	113 (23.01)	<0.001
History of CHD, *n* (%)	59 (7.07)	5 (1.45)	54 (11.00)	<0.001
History of stroke, *n* (%)	102 (12.22)	4 (1.16)	98 (19.96)	<0.001
White blood cell (10^9^/L)	7.00 (5.78–8.60)	6.71 (5.77–8.41)	7.18 (5.79–8.68)	0.110
Neutrophils (10^9^/L)	4.31 (3.26–5.92)	4.04 (3.11–5.63)	4.52 (3.47–6.09)	0.002
Platelets (10^9^/L)	232.00 (197.00–275.00)	232.00 (200.00–274.25)	233.00 (194.00–275.00)	0.533
Lymphocytes (10^9^/L)	1.74 (1.33–2.20)	1.87 (1.48–2.38)	1.65 (1.26–2.02)	<0.001
CRP (mg/L)	1.89 (0.50–4.13)	0.54 (0.50–2.50)	2.33 (0.53–5.00)	<0.001
Albumin (g/L)	39.20 (37.10–41.80)	40.10 (37.90–42.70)	38.80 (36.60–41.00)	<0.001
FPG (mmol/L)	5.21 (4.75–5.92)	5.06 (4.69–5.55)	5.39 (4.82–6.29)	<0.001
LDL-C (mmol/L)	3.14 (2.49–3.77)	3.29 (2.64–3.87)	3.05 (2.33–3.70)	<0.001
HDL-C (mmol/L)	1.14 (0.98–1.36)	1.22 (1.05–1.47)	1.09 (0.94–1.29)	<0.001
Homocysteine, μmol/L	10.50 (7.90–14.20)	8.50 (6.50–10.50)	12.70 (9.70–16.15)	<0.001
eGFR (mL/min × 1.73 m^2^)	96.08 (82.72–106.73)	102.38 (90.96–111.08)	91.27 (75.55–102.57)	<0.001
Uric acid, μmol/L	344.00 (282.50–423.50)	325.00 (269.75–389.25)	362.00 (292.00–437.50)	<0.001
LCR	1.09 (0.41–3.25)	2.40 (0.71–3.98)	0.73 (0.32–2.50)	<0.001

CSVD, cerebral small vessel disease; BMI, body mass index; SBP, systolic blood pressure; DBP, diastolic blood pressure; CHD history, coronary heart disease history; CRP, C-reactive protein; FPG, fasting plasma glucose; LDL-C, low-density lipoprotein cholesterol; HDL-C, high-density lipoprotein cholesterol; eGFR, estimated glomerular filtration rate; LCR, lymphocyte-to-C-reactive protein ratio.

### 3.2 Logistic regression analysis of factors associated with CSVD

Factors influencing CSVD were identified through logistic regression analysis (Table 3). Univariate analysis revealed significant associations (*p* < 0.05) between CSVD and factors of age, sex, SBP, smoking, alcohol consumption, hypertension, diabetes, coronary heart disease, stroke, NC, Alb, FPG, LDL-C, HDL-C, Hcy, eGFR, UA, and LCR. Furthermore, multivariate analysis indicated that age, SBP, smoking, hypertension, diabetes, coronary heart disease, stroke, FPG, Hcy, and LCR were independent risk factors for CSVD (*p* < 0.05).

**Table 3:** Results of the univariate and multivariate logistic regression analyses of factors affecting CSVD.

Variable	No. of Event/Median (IQR)	Univariate analysis		Multivariate analysis	
		OR (95%CI)	*P*-value	OR (95%CI)	*P*-value
Age (years)	63.00 (52.00–70.00)	1.10 (1.09–1.12)	<0.001	1.06 (1.03–1.08)	<0.001
Sex, *n* (%)					
Female	348 (41.68)	1(Ref)		1 (Ref)	
Male	487 (58.32)	2.39 (1.80–3.17)	<0.001	0.67 (0.39–1.16)	0.149
BMI (kg/m^2^)	23.46 (22.04–24.77)	1.02 (0.97–1.08)	0.407	0.93 (0.85–1.01)	0.081
SBP (mmHg)	137.00 (122.50–156.00)	1.04 (1.04–1.05)	<0.001	1.03 (1.02–1.04)	<0.001
Smoking status, *n* (%)					
Never	258 (30.9)	1 (Ref)		1 (Ref)	
Former and current	577 (69.1)	4.46 (3.14–6.34)	<0.001	4.25 (2.39–7.58)	<0.001
Drinking status, *n* (%)					
Never	109 (13.05)	1(Ref)		1 (Ref)	
Former and current	726 (86.95)	3.84 (2.29–6.43)	<0.001	0.94 (0.44–2.00)	0.879
Hypertension, *n* (%)	410 (49.1)	6.43 (4.71–8.78)	<0.001	2.19 (1.40–3.42)	<0.001
Diabetes, *n* (%)	124 (14.85)	9.05 (4.79–17.10)	<0.001	3.57 (1.44–8.89)	0.006
History of CHD, *n* (%)	59 (7.07)	8.38 (3.32–21.17)	<0.001	4.83 (1.57–14.88)	0.006
History of stroke, *n* (%)	102 (12.22)	21.20 (7.72–58.19)	<0.001	7.98 (2.58–24.71)	<0.001
Neutrophils (10^9^/L)	4.31 (3.26–5.92)	1.08 (1.01–1.15)	0.017	0.96 (0.87–1.06)	0.415
Albumin (g/L)	39.20 (37.10–41.80)	0.91 (0.88–0.95)	<0.001	0.97 (0.92–1.02)	0.250
FPG (mmol/L)	5.21 (4.75–5.92)	1.46 (1.29–1.66)	<0.001	1.27 (1.04–1.54)	0.017
LDL-C (mmol/L)	3.14 (2.49–3.77)	0.76 (0.65–0.87)	<0.001	0.85 (0.68–1.07)	0.175
HDL-C (mmol/L)	1.14 (0.98–1.36)	0.22 (0.14–0.36)	<0.001	0.64 (0.33–1.24)	0.186
Homocysteine, μmol/L	10.50 (7.90–14.20)	1.26 (1.21–1.32)	<0.001	1.12 (1.07–1.18)	<0.001
eGFR (mL/min × 1.73 m^2^)	96.08 (82.72–106.73)	0.96 (0.96–0.97)	<0.001	1.00 (0.98–1.01)	0.640
Uric acid, μmol/L	344.00 (282.50–423.50)	1.01 (1.01–1.01)	<0.001	1.00 (1.00–1.00)	0.838
LCR	1.09 (0.41–3.25)	0.72 (0.66–0.78)	<0.001	0.77 (0.69–0.87)	<0.001^*^

CSVD, cerebral small vessel disease; BMI, body mass index; SBP, systolic blood pressure; CHD history, coronary heart disease history; CRP, C-reactive protein; FPG, fasting plasma glucose; LDL-C, low-density lipoprotein cholesterol; HDL-C, high-density lipoprotein cholesterol; eGFR, estimated glomerular filtration rate; LCR, lymphocyte-to-C-reactive protein ratio.

* Adjusted for age, sex, BMI, SBP, smoking, drinking, hypertension, diabetes, history of CHD, history of stroke, neutrophils, albumin, FPG, LDL-C, HDL-C, homocysteine, eGFR, and uric acid.

### 3.3 Analysis of the relationship between LCR and CSVD

To further investigate the relationship between LCR and CSVD, we introduced categorical variables and continuous variables and constructed multivariate regression models (Table 4). After adjusting for potential confounders such as BMI and risk factors for CSVD in the categorical variable model, the odds ratios for Q2, Q3, and Q4 were 0.81 (95% CI = 0.44–1.48, *p* = 0.490), 0.56 (95% CI = 0.30–1.01, *p* = 0.054), and 0.29 (CI = 0.16–0.53, *p* < 0.001), respectively, compared with Q1, and there was a linear trend (*P*_trend_ < 0.001). Adjustments for potential confounders like BMI and risk factors for CSVD were also performed in the continuous variable model. The results revealed that the Log2-transformed LCR value was negatively associated with CSVD, with an OR of 0.78 (*p* < 0.001, 95% CI = 0.70–0.88).

**Table 4:** Association between LCR and the prevalence of CSVD in the participants.

Model	Categorical models					Continuous models	
	Q1 (0.22, ≤0.42)	Q2 (0.68, 0.42–1.09)	Q3 (2.31, 1.09–3.25)	Q4 (4.19, >3.25)	*P*_trend_	Log2-transformed LCR values	*P*-value
	(*n* = 211)	(*n* = 207)	(*n* = 209)	(*n* = 208)			
Model 1	1 (ref)	0.64 (0.39–1.04)	0.41 (0.25–0.66)	0.24 (0.15–0.39)	<0.001	0.75 (0.68–0.83)	<0.001
Model 2	1 (ref)	0.63 (0.36–1.10)	0.47 (0.27–0.81)	0.24 (0.14–0.41)	<0.001	0.76 (0.68–0.85)	<0.001
Model 3	1 (ref)	0.81 (0.44–1.48)	0.56 (0.30–1.01)	0.29 (0.16–0.53)	<0.001	0.78 (0.70–0.88)	<0.001

LCR, lymphocyte-to-C-reactive protein ratio; CSVD, cerebral small vessel disease.

Model 1: adjusted for age, and sex.

Model 2: adjusted for age, sex, BMI, smoking, drinking, hypertension, diabetes, history of CHD, and history of stroke.

Model 3: adjusted for age, sex, BMI, SBP, smoking, drinking, hypertension, diabetes, history of CHD, history of stroke, neutrophils, albumin, FPG, LDL-C, HDL-C, homocysteine, eGFR, and uric acid.

### 3.4 Analysis of the relationship between LCR and the common imaging markers of CSVD

We further analyzed the relationship between LCR and the common imaging markers of CSVD. In the categorical variable model, the severity of WMH, PWMH, DWMH, EPVS, BG-EPVS, and CSO-EPVS in the higher quartile groups had a significant linear trend (*P*_trend_ < 0.01) compared to the low quartile group, while the lacune showed a non-linear trend (*P*_trend_ = 0.096) (Table 5). The highest quartile (Q4) of LCR in the fully adjusted model (Model 3) was associated with lower WMH (OR = 0.45, 95% CI = 0.26–0.81, *p* = 0.007), PWMH (OR = 0.55, 95% CI = 0.31–0.97, *p* = 0.039), DWMH (OR = 0.51, 95% CI = 0.31–0.85, *p* = 0.010), EPVS (OR = 0.29, 95% CI = 0.17–0.48, *p* < 0.001), BG-EPVS (OR = 0.43, 95% CI = 0.24–0.75, *p* = 0.03), and CSO-EPVS burden (OR = 0.49, 95% CI = 0.29–0.82, *p* = 0.007). In the continuous variable model, after full adjustment in Model 3, LCR was negatively associated with WMH (OR = 0.84, 95% CI = 0.75–0.93, *p* = 0.001), PWMH (OR = 0.86, 95% CI = 0.77–0.96, *p* = 0.005), DWMH (OR = 0.88, 95% CI = 0.80–0.96, *p* = 0.005), EPVS (OR = 0.81, 95% CI = 0.74–0.89, *p* < 0.001), BG-EPVS (OR = 0.88, 95% CI = 0.80–0.97, *p* = 0.011), and CSO-EPVS (OR = 0.88, 95% CI = 0.81–0.96, *p* = 0.005). However, it was not associated with lacune (OR = 0.95, 95% CI = 0.86–1.04, *p* = 0.261).

**Table 5:** Association of LCR with the common imaging markers CSVD.

Model	Categorical models					Continuous models	
	Q1 (0.22, ≤0.42)	Q2 (0.68, 0.42–1.09)	Q3 (2.31, 1.09–3.25)	Q4 (4.19, >3.25)	*P*_trend_	Log2-transformed LCR values	*P*-value
	(*n* = 211)	(*n* = 207)	(*n* = 209)	(*n* = 208)			
Moderate to severe WMH burden							
Model 1	1 (ref)	0.65 (0.41–1.04)	0.44 (0.28–0.70)	0.33 (0.21–0.53)	<0.001	0.80 (0.73–0.87)	<0.001
Model 2	1 (ref)	0.67 (0.40–1.11)	0.52 (0.32–0.86)	0.37 (0.22–0.62)	<0.001	0.82 (0.74–0.90)	<0.001
Model 3	1 (ref)	0.92 (0.52–1.61)	0.60 (0.34–1.06)	0.45 (0.26–0.81)	0.002	0.84 (0.75–0.93)	0.001
Moderate to severe PWMH burden							
Model 1	1 (ref)	0.76 (0.48–1.19)	0.48 (0.31–0.76)	0.37 (0.23–0.59)	<0.001	0.81 (0.74–0.88)	<0.001
Model 2	1 (ref)	0.82 (0.50–1.34)	0.58 (0.36–0.95)	0.41 (0.25–0.69)	<0.001	0.83 (0.75–0.91)	<0.001
Model 3	1 (ref)	1.19 (0.68–2.08)	0.70 (0.40–1.21)	0.55 (0.31–0.97)	0.005	0.86 (0.77–0.96)	0.005
Moderate to severe DWMH burden							
Model 1	1 (ref)	0.71 (0.46–1.09)	0.54 (0.35–0.84)	0.41 (0.26–0.65)	<0.001	0.85 (0.78–0.92)	<0.001
Model 2	1 (ref)	0.72 (0.46–1.14)	0.63 (0.40–1.00)	0.46 (0.28–0.74)	0.002	0.87 (0.80–0.95)	0.001
Model 3	1 (ref)	0.83 (0.51–1.34)	0.64 (0.39–1.04)	0.51 (0.31–0.85)	0.007	0.88 (0.80–0.96)	0.005
Moderate to severe EPVS burden							
Model 1	1 (ref)	0.84 (0.55–1.28)	0.55 (0.36–0.84)	0.27 (0.17–0.43)	<0.001	0.81 (0.74–0.88)	<0.001
Model 2	1 (ref)	0.90 (0.57–1.41)	0.65 (0.41–1.01)	0.28 (0.17–0.47)	<0.001	0.82 (0.76–0.90)	<0.001
Model 3	1 (ref)	0.90 (0.56–1.44)	0.62 (0.38–0.99)	0.29 (0.17–0.48)	<0.001	0.81 (0.74–0.89)	<0.001
Moderate to severe BG-EPVS burden							
Model 1	1 (ref)	0.72 (0.46–1.14)	0.51 (0.32–0.82)	0.38 (0.23–0.64)	<0.001	0.87 (0.80–0.94)	<0.001
Model 2	1 (ref)	0.77 (0.48–1.24)	0.60 (0.37–0.98)	0.42 (0.24–0.72)	0.001	0.89 (0.81–0.97)	0.009
Model 3	1 (ref)	0.75 (0.46–1.24)	0.58 (0.35–0.97)	0.43 (0.24–0.75)	0.002	0.88 (0.80–0.97)	0.011
Moderate to severe CSO-EPVS burden							
Model 1	1 (ref)	1.22 (0.80–1.84)	0.78 (0.51–1.20)	0.43 (0.27–0.71)	<0.001	0.88 (0.81–0.95)	<0.001
Model 2	1 (ref)	1.30 (0.85–2.01)	0.89 (0.57–1.39)	0.46 (0.28–0.77)	<0.001	0.89 (0.82–0.96)	0.004
Model 3	1 (ref)	1.31 (0.83–2.05)	0.86 (0.54–1.37)	0.49 (0.29–0.82)	<0.001	0.88 (0.81–0.96)	0.005
Lacune							
Model 1	1 (ref)	0.84 (0.54–1.30)	0.60 (0.38–0.94)	0.50 (0.31–0.82)	0.003	0.90 (0.82–0.97)	0.008
Model 2	1 (ref)	0.95 (0.59–1.53)	0.77 (0.47–1.26)	0.59 (0.34–1.00)	0.034	0.93 (0.85–1.01)	0.095
Model 3	1 (ref)	1.08 (0.66–1.79)	0.79 (0.47–1.34)	0.70 (0.40–1.22)	0.096	0.95 (0.86–1.04)	0.261

LCR, lymphocyte-to-C-reactive protein ratio; CSVD, cerebral small vessel disease; WHM, white matter hyperintensity; PWMH, periventricular white matter hyperintensity; DWMH, deep white matter hyperintensity; EPVS, enlarged perivascular spaces; BG-EPVS, basal ganglia enlarged perivascular spaces; CSO-EPVS, centrum semiovale enlarged perivascular spaces.

Model 1: adjusted for age, and sex.

Model 2: adjusted for age, sex, BMI, smoking, drinking, hypertension, diabetes, history of CHD, and history of stroke.

Model 3: adjusted for age, sex, BMI, SBP, smoking, drinking, hypertension, diabetes, history of CHD, history of stroke, neutrophils, albumin, FPG, LDL-C, HDL-C, homocysteine, eGFR, and uric acid.

### 3.5 Dose-response relationship analysis

**Figure 3:**
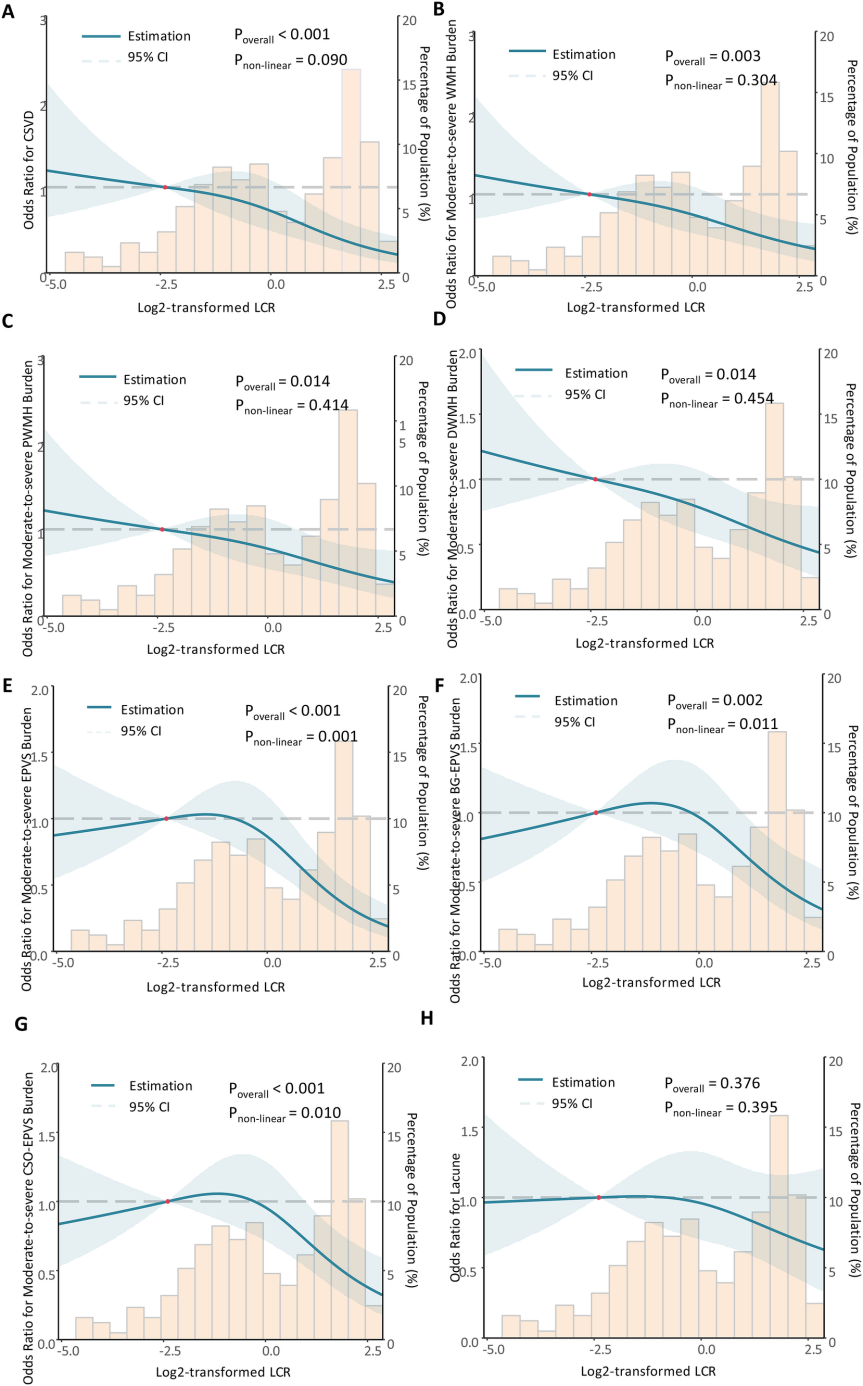
The dose-response relationship between LCR, CSVD, and its imaging markers. Restricted cubic spline analysis was conducted. The dose-response relationship between LCR and CSVD **(A)**, moderate-to-severe WMH **(B)**, moderate-to-severe PWMH **(C)**, moderate-to-severe DWMH **(D)**, moderate-to-severe EPVS **(E)**, moderate-to-severe BG-EPVS **(F)**, and moderate-to-severe CSO-EPVS **(G)**, and lacune **(H)** was shown. During restricted cubic spline analysis, the factors of age, sex, BMI, SBP, smoking status, alcohol consumption, hypertension, diabetes, coronary heart disease, stroke, NC, FPG, TC, LDL-C, HCY, eGRF, and UA were adjusted.

The dose-response relationship of LCR with CSVD and its imaging markers was analyzed using RCS (Figure 3). The corrected RCS model revealed a linear dose-response relationship between LCR and CSVD (*P*_non-linear_ = 0.090) (Figure 3A), moderate-to-severe WMH (*P*_non-linear_ = 0.304) (Figure 3B), moderate-to-severe PWMH (*P*_non-linear_ = 0.414) (Figure 3C), moderate-to-severe DWMH (*P*_non-linear_ = 0.454) (Figure 3D), and lacune (*P*_non-linear_ = 0.395) (Figure 3H). However, the dose-response relationship between LCR and moderate-to-severe EPVS (*P*_non-linear_ = 0.001) (Figure 3E), moderate-to-severe BG-EPVS (*P*_non-linear_ = 0.011) (Figure 3F), and moderate-to-severe CSO-EPVS (*P*_non-linear_ = 0.010) was non-linear (Figure 3G).

### 3.6 Subgroup analysis and interaction analysis

**Figure 4:**
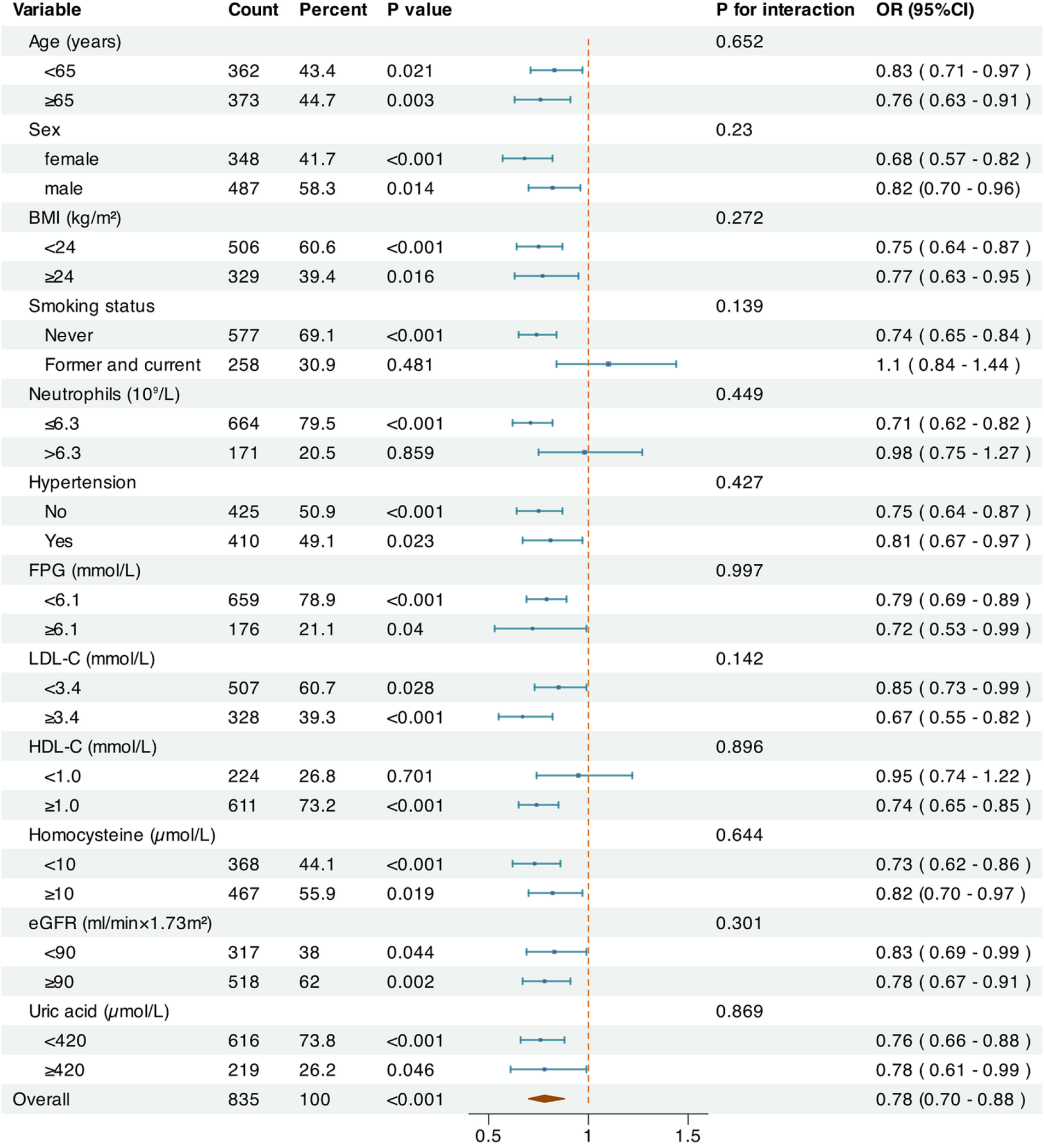
Subgroup analysis on the relationship between LCR and CSVD. The Log2-transformed value and the odds ratios for CSVD were used. The factors of age, sex, BMI, SBP, smoking status, alcohol consumption, hypertension, diabetes, coronary heart disease, stroke, NC, FPG, TC, LDL-C, HCY, eGRF, and UA were adjusted.

Multivariable logistic regression was performed in subgroups to evaluate the relationship between LCR and CSVD incidence after adjustment for other clinical confounders (Figure 4). Overall, LCR was significantly associated with CSVD across age, sex, BMI, history of hypertension, FPG, LDL-C, Hcy, eGFR, and UA subgroups (*p* < 0.05). However, statistical significance was observed only in non-smokers, those with NC ≤ 6.3 × 10^9^/L, and individuals with HDL-C ≥ 1 mmol/L among the subgroups of smoking history, NC and HDL-C. In addition, no significant interactions were detected between LCR and the variables in the interaction analysis (*P* for interaction >0.05).

### 3.7 The predictive value of LCR and model 3 for CSVD

**Figure 5:**
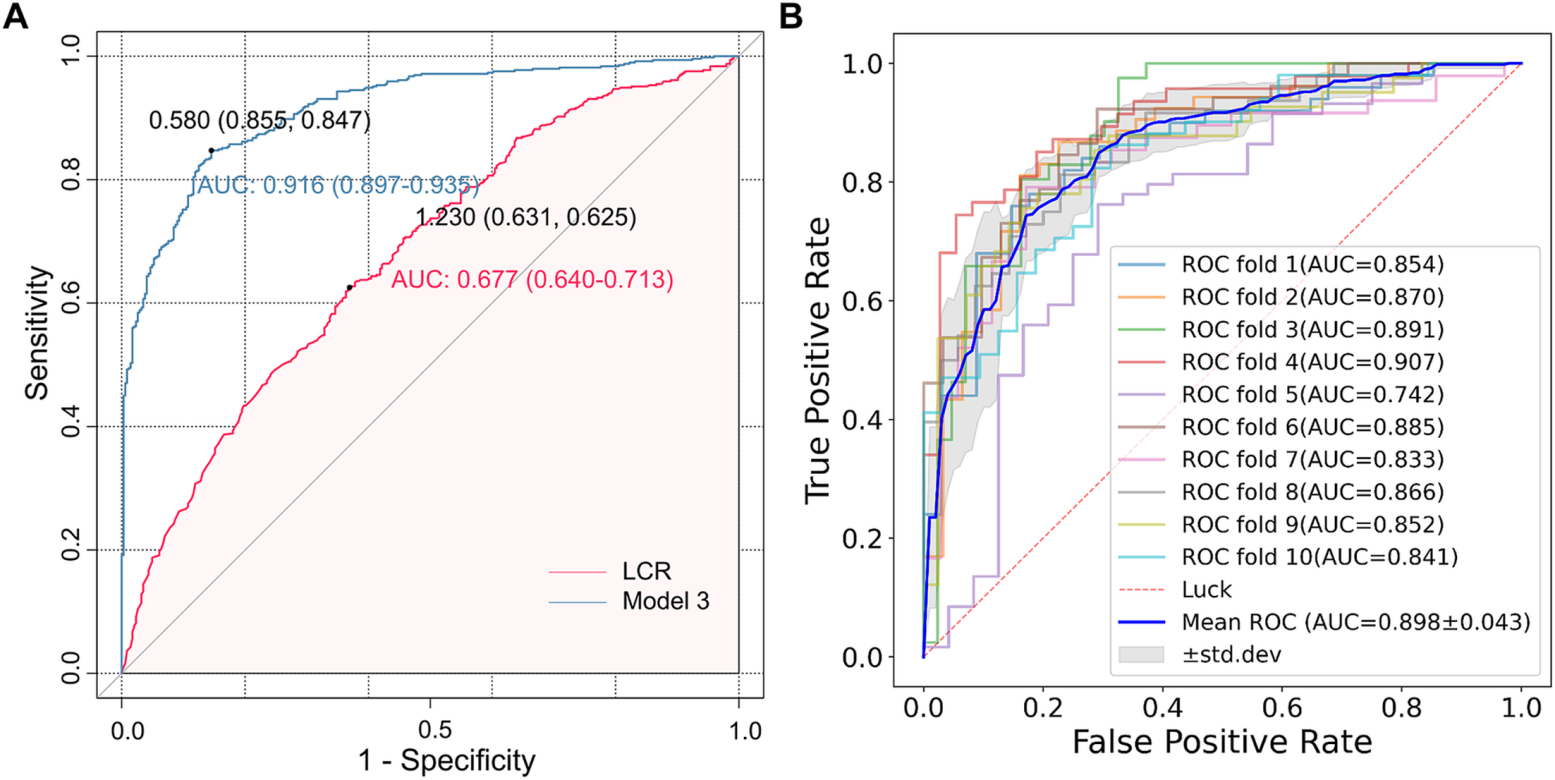
The ROC analysis of LCR for predicting CSVD. **(A)** The AUC values of LCR and Model 3 were 0.677 (0.640–0.713) and 0.916 (0.897–0.935), respectively, and the optimal cut-off values were 1.230 and 0.580, respectively. **(B)** The AUC of the average ROC curve was 0.898 ± 0.043, and the AUC of each fold was also presented in the figure.

The ROC curve analysis showed that the AUC of LCR for predicting CSVD was 0.677 (95% confidence interval 0.640–0.713, *p* < 0.001) (Figure 5A). The optimal cut-off value for LCR was determined to be 1.230. At this optimal cut-off, the sensitivity for predicting CSVD was 63.1%, and the specificity was 62.5%. Furthermore, the AUC of Model 3 for predicting CSVD was 0.916 (95% CI = 0.897–0.935). The optimal cut-off value for LCR was determined to be 0.580. At this optimal cut-off, the sensitivity for predicting CSVD was 89.7%, and the specificity was 93.5%. Additionally, the cross-validated mean AUC of Model 3 was 0.898 ± 0.043 (Figure 5B). The accuracy, precision, F1-score, and recall of the calculated average ROC curve were 0.827, 0.845, 0.855, and 0.865, respectively. These results demonstrate that LCR and Model 3 present a good predictive performance.

## 4 Discussion

In this cross-sectional study, we examined the association of LCR with CSVD and its common imaging markers. Data were collected from 835 participants with or without CSVD. We observed a significantly higher incidence of CSVD in the low quartile of LCR than in the high quartile, and a greater burden of WMH, EPVS, and lacune. Multivariate regression analysis revealed that elevated levels of LCR were independently associated with a reduced risk of CSVD, suggesting that LCR is a protective factor for CSVD. Furthermore, a negative association was found between LCR and moderate-to-severe WMH and EPVS. However, LCR was not significantly associated with lacune. In the RCS analysis, we found a linear dose-response relationship of the log-transformed LCR with the occurrence of CSVD and moderate-to-severe WMH, but a non-linear relationship with moderate-to-severe EPVS. Additionally, LCR remained associated with CSVD in most subgroups. However, the effectiveness of the test may be limited due to the small sample size. In the subgroups of smoking, NC, and HDL-C, the correlation between LCR and CSVD was significant only in non-smokers, those with NC ≤ 6.3 × 10^9^/L, and individuals with HDL-C ≥ 1 mmol/L. Interaction analysis indicated the absence of interaction effects between each variable and LCR. Additionally, the ROC curve analysis found that LCR had good performance in predicting CSVD. Overall, our results suggest a role for LCR in evaluating the pathogenesis and severity of CSVD.

CRP is an acute-phase protein synthesized in the liver in response to proinflammatory stimuli like IL-6 and acts as a sensitive, albeit not specific, indicator of inflammation. A meta-analysis (38) revealed a log-linear relationship between CRP levels and the risk of death from ischemic stroke in a healthy population. Furthermore, the low-level inflammatory response detected by high-sensitivity CRP in patients with minor strokes was also a risk factor for stroke recurrence (39). In this study, we detected full-range CRP, which includes both standard and high-sensitivity CRP, thereby capturing a wide range of CRP levels. This approach further enhances our understanding of the relationship between extensive inflammatory responses and CSVD.

Lymphocytes play a crucial role in the immune response and can secrete anti-inflammatory cytokines such as IL-10 to promote repair and protect against arteriosclerosis and brain damage (40). Previous studies have demonstrated that lower peripheral lymphocyte proportions independently predict adverse outcomes following cerebrovascular events (41, 42). LCR is calculated by dividing the lymphocyte count by the CRP concentration, serving as an indicator of systemic inflammation. A decline in LCR may suggest a reduction in adaptive immunity and an increase in inflammation levels. To our knowledge, this is the first study to reveal the association between LCR and cerebrovascular disease. Previously, LCR has been primarily used to assess malignancies (43) and some cardiovascular conditions (44). A multicenter study (45) found an association between LCR and survival in patients with cancer-related malnutrition, demonstrating an inverse L-shaped dose-response relationship. LCR has predictive value for cardiovascular adverse events in patients with ST-segment elevation myocardial infarction after surgery, wherein a high preoperative LCR acts as an independent protective factor against major adverse cardiovascular events both in the hospital and during follow-up (26). In addition, LCR is an outcome predictor for patients undergoing cardiac surgery, and low levels of LCR are associated with higher all-cause mortality (46). Another study found that among patients with acute ischemic stroke due to anterior circulation large vessel occlusion who received endovascular treatment, the LCR levels in the futile recanalization group were significantly lower, suggesting that LCR may act as a protective factor for cerebrovascular diseases (47). Our results further enhanced the understanding of the association between LCR and CSVD. After adjusting for age, sex, BMI, SBP, history of hypertension, history of diabetes, history of coronary heart disease, history of stroke, NC, LDL-C, HDL-C, albumin, FPG, Hcy, eGFR, UA, etc., we found a negative association between LCR and CSVD, suggesting that LCR may be an independent protective factor of CSVD. A possible mechanism is that in patients with CSVD, there may be immune system degeneration, specifically a weakening of the adaptive immune response. This weakening results in reduced circulating lymphocytes, which may display characteristics of senescent cells. Senescent immune cells then generate a pro-inflammatory senescence-associated secretory phenotype, further exacerbating inflammation and promoting disease progression (48–50).

The primary inflammatory mechanisms in CSVD involve the following pathways: (1) endothelial dysfunction is recognized as a crucial factor in CSVD pathophysiology. Reduced activity of endothelial nitric oxide synthase results in diminished nitric oxide synthesis and bioavailability, along with a significant rise in reactive oxygen species in endothelial cells. This oxidative stress can cause DNA damage, increased release of inflammatory mediators, widespread inflammation, and apoptosis (51, 52). (2) Furthermore, disruption of the blood–brain barrier represents a critical initial alteration in brain small-vessel disease. Activation of microglia in ischemic brain regions triggers the generation of matrix metalloproteinases. These enzymes can degrade the basement membrane and disrupt the tight junctions between endothelial cells, disrupting blood–brain barrier permeability. This disruption not only facilitates the entry of inflammatory cells from the blood into brain tissue but also triggers the release of pro-inflammatory mediators by endothelial cells in the microcirculation, secreting various adhesion molecules like intercellular adhesion molecule-1 and vascular cell adhesion molecule-1. These molecules promote leukocyte recruitment, thereby amplifying the procoagulant function and proinflammatory response at the vascular injury site (53, 54). (3) In addition, vascular risk factors, such as hypertension, can induce persistent vascular inflammatory responses that contribute to pathological arteriosclerosis alterations. These changes sustainably decrease vascular perfusion, activating hypoxia-sensitive genes (e.g., hypoxia-inducible factor-1α) and provoking molecular cascades that exacerbate vascular damage (55, 56).

Our study further examined the relationship between LCR and common imaging markers of CSVD. Firstly, we evaluated the relationship between LCR and WMH severity. Nam et al. reported that elevated NLR was associated with increased WMH volumes in a healthy population, suggesting that the brain WMH burden in high-risk populations may be predicted through assessing NLR (57). Consistently, we found that LCR was independently associated with moderate to severe WMH burden and this association remained significant in subsequent regional analyses. There was a linear dose-response relationship between LCR and WMH, suggesting the potential involvement of chronic inflammation and inherited immunity in the pathogenesis of WMH, with a proportional rise in the risk of severe WMH burden corresponding to the Log2-transformed LCR values. Secondly, we assessed the association between LCR and the EPVS burden in different brain regions. Inflammatory markers tend to gather around damaged cerebral vessels, while inflammatory cells accumulate within the PVS, leading to alterations and remodeling of fluid clearance, which in turn leads to an increase in PVS volume (58). Previous studies have indicated that in patients with systemic lupus erythematosus, systemic inflammation levels are associated with EPVS, particularly CSO-EPVS, but not BG-EPVS (59). In contrast, our results showed that both CSO-EPVS and BG-EPVS were significantly associated with LCR, exhibiting an inverse L-shaped dose-response relationship. One possible explanation is that the EPVS burden in different brain regions may represent different subtypes of CSVD. Previous studies (60, 61) have shown that CSO is prone to cerebral amyloid angiopathy, while BG is more closely associated with deep perforating arteritis. It is hypothesized that cerebral amyloidosis in CSO may trigger T-cell infiltration, which in turn leads to a reduction in circulating lymphocytes and a subsequent decrease in LCR (62, 63). However, due to simultaneous interference with local antigen presentation and T cell activation, cerebral immune surveillance fails to orchestrate an effective immune response against amyloid beta, which results in amyloid beta accumulation and subsequent PVS expansion (62). Endothelial and blood–brain barrier dysfunction are pivotal in the onset of deep perforating arteritis. The decrease in LCR leads to a reduction in endothelial tight junctions and protein leakage into the perivascular space, ultimately leading to BG-EPVS (64). Finally, we conducted an assessment of the relationship between LCR and lacune. A previous cohort study found that lacune was significantly correlated with vascular/endothelial dysfunction-related inflammatory markers, such as Hcy, but not with systemic inflammatory markers, such as NLR (65). Consistently, our results revealed that there was no independent association between LCR and lacune. We speculate that lacune may be more closely associated with the mechanisms of vascular endothelial dysfunction.

Additionally, subgroup analyses revealed that the association between LCR and CSVD remained in most subgroups. Subsequent interaction analyses showed no interaction effect between the variables and LCR. Nevertheless, among the smoking, NC, and HDL-C subgroups, the significant correlation between LCR and CSVD was evident only in non-smokers, with NC ≤ 6.3 × 10^9^/L, and HDL-C ≥ 1 mmol/L. This limited significance could be attributed to the lack of test efficacy due to the small sample size, warranting further investigation in future studies.

The LCR serves as a composite biomarker that captures the relationship between inflammation and immune response, which is particularly relevant in the context of CSVD. While traditional metrics such as CRP alone indicate systemic inflammation and other clinical markers like the NLR provide insights into leukocyte dynamics, the LCR specifically reflects the balance between the adaptive immune response (as represented by lymphocyte levels) and inflammation (as indicated by CRP levels) (66–70). This unique perspective allows for a more nuanced understanding of the inflammatory processes involved in CSVD. Moreover, the LCR can enhance risk stratification and patient management by providing additional information regarding immune function in the context of chronic inflammation associated with vascular health, compared with metrics like hs-CRP (71). Therefore, incorporating LCR into clinical practice may improve the predictive accuracy for CSVD severity and guide interventions. Rather than replacing existing metrics used to evaluate CSVD, we propose that the LCR can be used in combination with these established markers. Given the simplicity and cost-effectiveness of calculating the LCR from routine blood tests, it serves as an accessible tool for clinicians. Its integration into routine laboratory assessments could assist in identifying patients at risk for more severe forms of CSVD, such as minor stroke, facilitating timely intervention and personalized management strategies.

This study has some limitations. First of all, this is a single-center cross-sectional study. There may be potential selection bias. Second, inflammatory markers frequently interact within various pathways, indicating that composite markers may have higher sensitivity. Future investigations could encompass a broader array of potential CSVD biomarkers for comprehensive evaluation. In addition, we only studied baseline levels of inflammatory markers and imaging, neglecting their dynamic nature throughout the disease progression. Therefore, further longitudinal studies are warranted to validate our findings.

In conclusion, our findings revealed that LCR was an independent protective factor for CSVD, associated with lower WMH and EPVS burden, but not lacune. The study highlighted the potential role of inflammation in the pathophysiology of CSVD through various pathways. The linear dose-response relationship between LCR and CSVD as well as moderate-to-severe WMH was observed, indicating a potential link between chronic inflammation and CSVD. Subgroup analyses further supported the association of LCR with CSVD in most subgroups, with notable correlations found in non-smokers, those with specific neutrophil counts, and individuals with HDL-C levels. Moreover, interaction analyses indicated no significant interaction effects, suggesting the independent role of LCR in CSVD. In addition, the ability of LCR to predict CSVD, as demonstrated by our ROC curve analysis, further highlights its utility as a potential clinical biomarker for early detection and monitoring of disease progression. These results contribute to a deeper understanding of the pathogenesis of CSVD and suggest inflammation as a potential target for intervention in the future. Further studies are warranted to validate these findings and explore the dynamic nature of inflammatory markers in CSVD progression.

## Data Availability

The raw data supporting the conclusions of this article will be made available by the authors, without undue reservation.
